# Malate synthase contributes to the survival of *Salmonella* Typhimurium against nutrient and oxidative stress conditions

**DOI:** 10.1038/s41598-022-20245-0

**Published:** 2022-09-25

**Authors:** Ratanti Sarkhel, Shekhar Apoorva, Swagatika Priyadarsini, Hari Balaji Sridhar, Sanjeev Kumar Bhure, Manish Mahawar

**Affiliations:** Division of Biochemistry, ICAR-Indian Veterinary Research Institute, Izatnagar, 243122 India

**Keywords:** Bacteriology, Biochemistry, Biological techniques, Microbiology

## Abstract

To survive and replicate in the host, *S. *Typhimurium have evolved several metabolic pathways. The glyoxylate shunt is one such pathway that can utilize acetate for the synthesis of glucose and other biomolecules. This pathway is a bypass of the TCA cycle in which CO_2_ generating steps are omitted. Two enzymes involved in the glyoxylate cycle are isocitrate lyase (ICL) and malate synthase (MS). We determined the contribution of MS in the survival of *S. *Typhimurium under carbon limiting and oxidative stress conditions. The *ms* gene deletion strain (∆*ms* strain) grew normally in LB media but failed to grow in M9 minimal media supplemented with acetate as a sole carbon source. However, the ∆*ms* strain showed hypersensitivity (*p* < 0.05) to hypochlorite. Further, ∆*ms* strain has been significantly more susceptible to neutrophils. Interestingly, several folds induction of *ms* gene was observed following incubation of *S.* Typhimurium with neutrophils. Further, ∆*ms* strain showed defective colonization in poultry spleen and liver. In short, our data demonstrate that the MS contributes to the virulence of *S.* Typhimurium by aiding its survival under carbon starvation and oxidative stress conditions.

## Introduction

Based on antigenic presentations^1^, *Salmonella enterica* serovars are grouped as typhoidal and non-typhoidal *Salmonella* (NTS). WHO recognizes NTS as one of the three most common food-borne bacterial diseases in humans all over the world. Old, young, and immunocompromised individuals are highly prone to *Salmonella* infection^2^. Among NTS, serovar Typhimurium is most commonly isolated from patients around the globe^3^.

Following ingestion, a proportion of the microorganisms resists the low gastric pH, invades the intestinal mucosa, and replicates in the sub-mucosa and Peyer’s patches^4^. Following intestinal penetration, *S.* Typhimurium gains access to the mesenteric lymph nodes, where the bacteria are engulfed by phagocytic cells, such as macrophages^5^. Once inside the macrophages, *S*. Typhimurium is compartmentalized into a modified vacuole known as “*Salmonella*-containing vacuole” (SCV) and represents a central feature in the intracellular survival and growth of this bacterium^6^. Thus, the engulfment by the macrophage thrusts the *S.* Typhimurium into an alien milieu which is rich in various antimicrobials and devoid of key nutrients essential for metabolism and replication. To survive under such harsh conditions, *S*. Typhimurium modulates the functions of phagocytes in several ways. First, effectors encoded by the type III secretion system of *S*. Typhimurium impede the assembly of phagosomal oxidase and consequently inhibit the production of superoxide radicals. Second, the SCV acts as a shield for *S.* Typhimurium that not only prevents lysosomal fusion but also limits the exposure of contained bacterial cells to antimicrobial agents^7^. While the primary antioxidants of *S.* Typhimurium directly quench oxidants, the repair enzymes restore the functions of the damaged biomolecules^8,9^.

However, survival against the antimicrobial assault in the phagolysosome depends on the microbe’s ability to synthesize the proteins and other biomolecules required to counteract stresses. Thus, a pathogen must find the requisite nutrients to provide the building blocks for these complex macromolecules and the energy with which to synthesize them^10^. It is the metabolic flexibility of *S.* Typhimurium which allows it to survive in such harsh conditions within the host^11^. The ability to fulfill its nutrient requirements from alternate sources might play an important role in the adeptness of *S.* Typhimurium in the host. One such survival mechanism is the existence of the glyoxylate cycle, whose primary function is to permit bacterial/cellular growth when C_2_ compounds, such as ethanol and acetate, are the only sources of carbon^12^. Few studies suggest that macrophages are rich in fatty acids. Upon metabolism, fatty acids generate acetyl-CoA that can be converted to acetate^13^, a substrate for the glyoxylate cycle.

The glyoxylate shunt consists of six of the eight reactions of the TCA cycle but bypasses the two oxidative steps in which carbon dioxide is evolved^14^. The two unique enzymes of the glyoxylate cycle are isocitrate lyase (ICL encoded by *aceA*^15^ and malate synthase (MS encoded by *aceB*^16^). While ICL catalyzes the cleavage of isocitrate to succinate and glyoxylate, MS condenses glyoxylate with an acetyl group from acetyl-CoA to produce malate. The net result of the glyoxylate cycle is the production of malate and succinate from two molecules of acetyl-CoA derived from acetate or the degradation of ethanol, fatty acids, or poly-β-hydroxybutyrate^17^.

The potential contribution of the glyoxylate cycle in the survival of microorganisms under oxidative stress and pathogenesis has been suggested. Few studies in fungal and bacterial pathogens have indicated the upregulation of glyoxylate cycle genes following phagocytosis. An increased metabolic flux (48%) through the glyoxylate shunt is observed in *E. coli* experiencing superoxide stress^18^. A recent study in *M. tuberculosis* showed the increased susceptibility of *ms* knockdown strain to oxidative and nitrosative stresses, and macrophages^19^. It has been suggested that ICL is required for the persistence of *Salmonella* during chronic infection in mice^20^.

Earlier, we have shown that the *ms* gene deletion strain showed defective colonization in poultry caecum^9^. Further, Met residues of MS are prone to oxidation and can be repaired by methionine sulfoxide reductase. However, the role of MS in the survival of *S.* Typhimurium under oxidative stress conditions is not known. In the present study, we aimed to explore the contribution of *ms* in the survival of *S.* Typhimurium during oxidative stress and colonization in the liver and spleen of poultry. By employing various biochemical, molecular biology tools along with cell culture and live animal studies we show that the *ms* is required for growth of *S.* Typhimurium under carbon starvation and survival under oxidative stress conditions. Finally, our data suggest that the *ms* contributes to the survival of *S.* Typhimurium in phagocytic cells and poultry spleen and liver.

## Results

### Δ*ms* strain does not exhibit defective growth in LB broth

**Figure 1:**
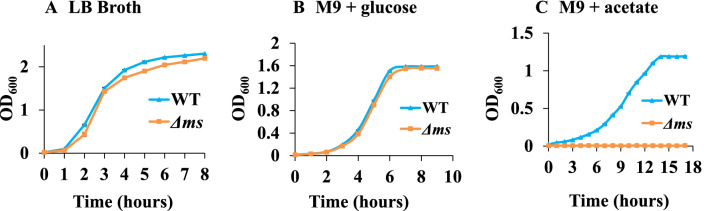
Δ*ms* strain failed to grow on acetate as a sole carbon source : overnight grown cultures of WT and Δ*ms* strains of *S.* Typhimurium were diluted in LB broth (**A**) or M9 media supplemented with either 0.4% glucose (**B**) or 0.4% acetate (**C**) and grown on a shaker incubator. The aliquots were taken at an interval of 1 h and optical densities were measured at 600 nm. Data is presented as mean ± SD (n = 3).

The growth rate of Δ*ms* strain was comparable to that of *S*. Typhimurium in LB broth. However, the Δ*ms* strain failed to grow in M9 media supplemented with acetate as the sole carbon source (Fig. 1 and Supplementary Fig. 1). Further, we observed the induction of malate synthase protein in *S.* Typhimurium cultured in the M9 media supplemented with acetate (Supplementary Fig. 2). The immunoreactivity of MS band was highly intense in the lysates of *S*. Typhimurium grown in M9 minimal media supplemented with acetate as compared to bacteria cultured in glucose added media (Supplementary Fig. 2a). However, the loading on SDS-gel was almost similar for both the conditions (Supplementary Fig. 2b).

### Malate synthase is susceptible to oxidative damage

Earlier, we have reported oxidant mediated oligomerization and Met-SO formation in malate synthase^9^. To corroborate our findings, if malate synthase is prone to oxidation, we assessed the carbonylation by Oxyblot. Carbonylation is a well-accepted marker to assess protein oxidation^21^. Our Oxyblot analysis showed a much more intense band in oxidant exposed malate synthase as compared to control (Supplementary Fig. 3).

### Δ*ms* strain shows hypersusceptibility to HOCl

**Figure 2:**
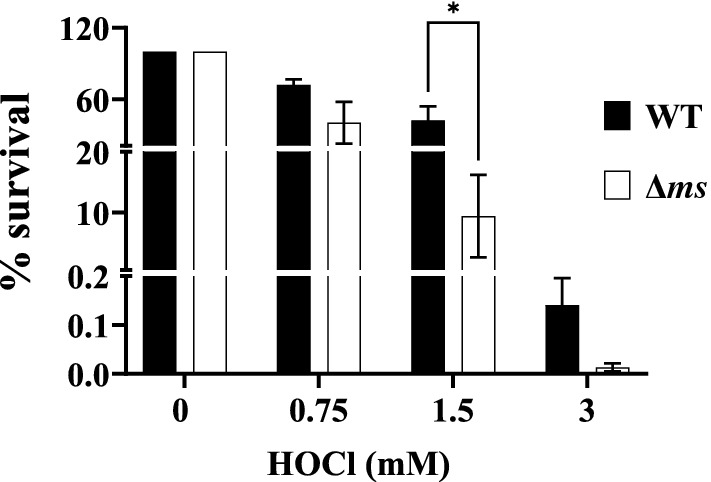
Δ*ms* strain of *S*. Typhimurium is hypersensitive to HOCl: WT and Δ*ms* strains of *S*. Typhimurium were grown in LB broth up to late stationary phase. The cultures were then exposed to indicated concentrations of NaOCl for 2 h. Following exposure, the cultures were serially diluted and plated on HEA plates. Colonies were enumerated following incubation of the plates, expressed in percentage of recovered viable cells. Results are shown as mean ± SE (n = 3) and is representative of two experiments. * *p* < 0.05.

HOCl is one of the most potent oxidants generated by myeloperoxidase catalyzed reaction between H_2_O_2_ and chloride ions. We assessed the susceptibility of Δ*ms* strain to HOCl. The percent recovery of the Δ*ms* strain was found to be reduced up to 9.39% and 0.013% as compared to that of 42.31% and 0.141% in the case of WT after treating the cells with 1.5 and 3 mM HOCl respectively (Fig. 2).

### Δ*ms* strain is highly susceptible to neutrophils

**Figure 3:**
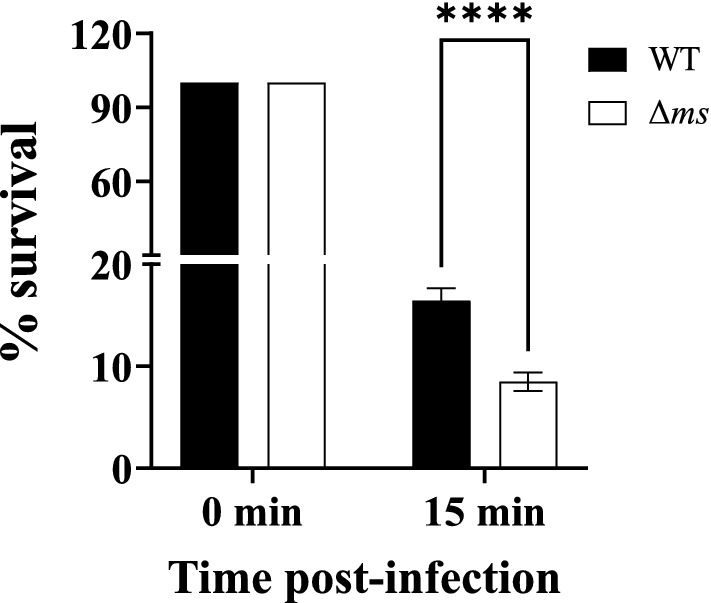
Δ*ms* strain shows hypersensitivity to neutrophils: the neutrophils were infected with WT and Δ*ms* strains of *S.* Typhimurium @ MOI of 10:1 (bacteria:cells). Following 15 min of incubation, the neutrophils were lysed by Triton X-100. The lysates were serially diluted and plated on HE agar plates. Colonies were enumerated following incubation of the plates. Comparison of percentage of recovered WT and Δ*ms* strains of *S.* Typhimurium after 0 min and 15 min post-infection is shown. Data are presented as mean ± SE (n = 5). ****p < 0.0001.

Neutrophils are the key HOCl producing cells. After observing susceptibility of Δ*ms* strain to reagent HOCl, we investigated the role of *ms* gene in the survival of *S.* Typhimurium against neutrophil-mediated killing. Neutrophils were incubated with WT or Δ*ms* strains of *S.* Typhimurium. By retrospective plating, the observed MOI for WT and Δ*ms* strains were 1:7 and 1:8.5 (neutrophil: bacteria), respectively. Following 15 min of incubation, the percent recovery of the Δ*ms* strain was found to be reduced up to 8.47% as compared to that 16.42% in case of WT after incubation with neutrophils (Fig. 3). Indeed, the Δ*ms* strain was significantly more susceptible (*p* < 0.0001) to neutrophil mediated killing.

### Incubation of *S*. Typhimurium with neutrophil induces expression of malate synthase

After observing the hypersusceptibility of Δ*ms* strain to neutrophils, we evaluated the expression of *ms* following incubation of *S.* Typhimurium with neutrophils. The *S.* Typhimurium was co-cultured with neutrophils for 15 min and the relative expression of *ms* gene was analyzed by qRT-PCR. We observed 3.82 folds upregulation of *ms* in *S.* Typhimurium following incubation with neutrophils (Supplementary Fig. 6).

### Δ*ms* strain is attenuated in spleen and liver

**Figure 4:**
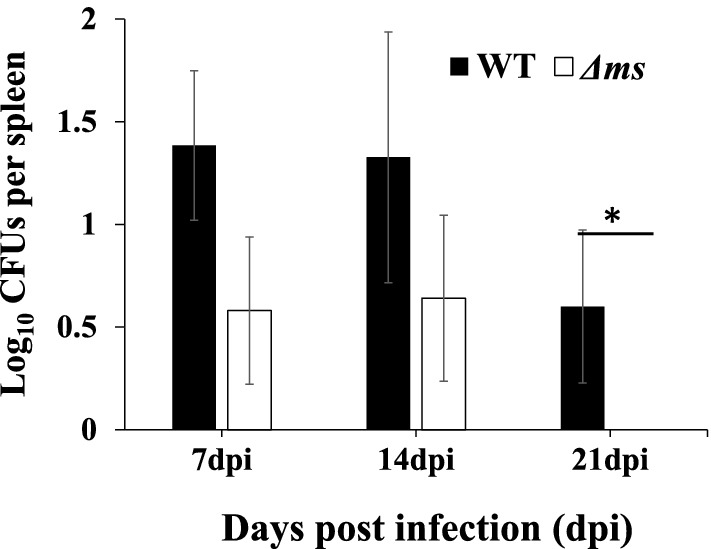
Quantitation of bacterial burden in the spleen upon oral infection of birds with WT or Δ*ms* strains of *S.* Typhimurium ***: ***the spleen was homogenized in sterile PBS and 100 μl homogenate was plated on HEA plates. The CFU per spleen was calculated after 7 and 14 days post infection (dpi). The data are presented as mean ± SE (n = 5). * *p* < 0.05.

Poultry is one of the most important reservoirs of *S.* Typhimurium in nature which transmits infection through eggs and meat. After a short enteric phase, *S.* Typhimurium enters the spleen and liver where it targets various phagocytic cells. We determined the bacterial loads in the poultry spleen and liver on 7, 14 and 21 days post-infection. In the spleen of WT strain infected birds, we obtained bacteria at all times post-infection. The numbers of *S.* Typhimurium recovered on 7, 14 and 21 days were expressed in log_10_ CFU/spleen (mean ± SE). Number of WT strain recovered were 1.4 ± 0.36, 1.3 ± 0.61 and 0.6 ± 0.37 respectively. However, the survival of ∆*ms* strain was compromised in the spleen and we recovered mutant bacteria only up to 14 days post-infection. The counts were 0.58 ± 0.36 and 0.64 ± 0.4 on days 7 and 14th respectively (Fig. 4).

**Figure 5:**
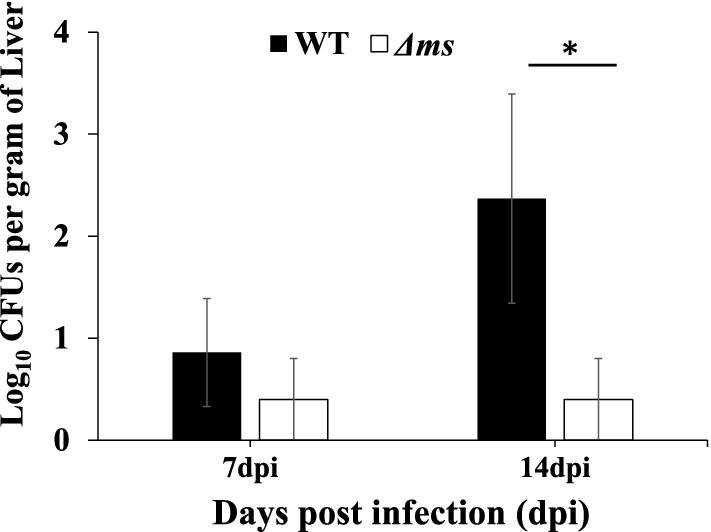
Analysis of bacterial burden in the liver upon oral infection of birds with WT or Δ*ms* strains of *S.* Typhimurium : the liver (100 mg) was homogenized in sterile PBS and 100 μl homogenate was plated on HEA plates. The CFU per gram of liver was calculated after 7 and 14 days post infection (dpi). The data are presented as mean ± SE (n = 5). *p < 0.05.

In the liver, we recovered bacteria from both WT and ∆*ms* strain infected birds until 14 days post-infection and expressed the values as log_10_ CFU/ gm of the liver (mean ± SE). The numbers of WT bacteria recovered were 0.86 ± 0.5 and 2.37 ± 1.0 on 7 and 14 days post-infection, respectively. In ∆*ms* strain infected birds, we recovered 0.4 ± 0.4 and 0.4 ± 0.4 on 7th and 14th post-infection, respectively (Fig. 5). On 14 days post-infection the bacterial loads in the liver of ∆*ms* strain infected birds were significantly (*p* < 0.05) lower than the WT-infected group.

## Discussion

To thrive within the host, *S*. Typhimurium has to confront nutrient/ carbon limiting and oxidant rich environment of the phagocytic cells. To assess the role of the glyoxylate cycle in *S*. Typhimurium under these two (carbon starvation and oxidative) stress conditions, we deleted the malate synthase gene and confirmed the deletion by PCR^9^. Δ*ms* strain grew normally in LB media or M9 minimal media supplemented with glucose (Fig. 1A,B and Supplementary Fig. 1). However, Δ*ms* strain displayed total growth defect when cultured in M9 minimal media supplemented with acetate as a sole carbon source (Fig. 1C and Supplementary Fig. 1).

Induction of glyoxylate shunt enzymes has been reported in *S*. Typhimurium cultured in acetate containing media^22^. We observed huge (more than 20 folds) induction of malate synthase protein in *S*. Typhimurium grown in M9 minimal media supplemented with acetate as compared to cultured in M9 minimal media supplemented with glucose (Supplementary Fig. 2). These data suggest a pivotal role of malate synthase in the survival of *S*. Typhimurium under carbon limiting conditions.

Under oxidative stress, the activation of the enzymes of the glyoxylate shunt has been observed in various microorganisms including, *Pseudomonas aeruginosa*^23^, *M. tuberculosis*^24^, *Alishewanella*^25^ and *Acinetobacter oleivorans*^26^. Bioinformatics analysis suggests that the microorganisms in which the glyoxylate cycle is functional, are either aerobic or facultative anaerobic^23^. The use of oxygen to oxidize nutrients and to obtain energy through respiration generates superoxides, hydrogen peroxide, and the highly reactive hydroxyl radicals^18^. Thus, microorganisms encounter potentially lethal levels of these ROS due to oxidative burst of phagosomes as well as from their normal aerobic metabolism^27^. In fact, NADH primarily serves for the generation of ATP, however, its oxidation during aerobic respiration is responsible for the generation of most of the endogenous ROS^28^. Increased metabolite flow through the glyoxylate shunt instead of the TCA cycle reduces the amount of NADH production from glucose. The reduction in NADH generation decreases total ROS as superoxides generated from cellular metabolism will be minimal^18^. Further, in contrast to eukaryotes, isocitrate dehydrogenase (IDH) in bacteria that are capable to grow in acetate is linked to NADP rather than NAD. During oxidative stress, NADH functions as the major nicotinamide nucleotide reductant^28^. Due to greater reactivity with Fe^3+^ generated by Fenton’s reaction, the NADH levels rapidly get depleted in comparison to NADPH. In agreement with the above hypothesis paraquat stressed *E. coli* showed increased production of acetate and flux in the glyoxylate cycle consequently increased NADPH:NADH ratio^18^. Hence, the NADP dependence of bacterial IDHs appears to be an adaptation to grow on acetate wherein the glyoxylate cycle diminishes the level of NADH without jeopardizing its upregulation by NADP mediated phosphorylation of IDH. These interesting insights indicate the potential contribution of the glyoxylate cycle in the survival of microorganisms under oxidative stress.

This prompted us to examine the effect of *ms* gene deletion on the survival of *S*. Typhimurium under both carbon starvation and oxidative stress conditions. To mimic these two conditions, we exposed nutrient-deprived cells (grown-up to late stationary phase) to HOCl. Δ*ms* strain has been more susceptible (*p* < 0.05) to HOCl as compared to WT strain of *S*. Typhimurium (Fig. 2), suggesting an important role of malate synthase/ glyoxylate cycle in the survival of *S*. Typhimurium under oxidative stress. Malate synthase gene deletion strain of *P*. *aeruginosa* showed hypersusceptibility to paraquat, a chemical oxidant^23^. A different study demonstrated the hypersensitivity of mutants of *P*. *aeruginosa* in glyoxylate cycle enzymes to H_2_O_2_^29^.

Neutrophils are the key HOCl producing cells. Thus, we evaluated the role of *ms* in the survival of *S*. Typhimurium against neutrophils. In comparison to WT strain of *S*. Typhimurium, Δ*ms* strain has been highly susceptible (*p* < 0.0001) to neutrophils (Fig. 3). Glyoxylate cycle enzymes contribute to the survival of various pathogens against phagocytic cells. *icl* deficient strain of *Rhodococcus equi*^30^, *ms* knockdown strain of *M. tuberculosis*^19^ showed defective survival following exposure to phagocytic cells.

After observing the hypersusceptibility of Δ*ms* strain to neutrophils, we wondered if *ms* gene gets induced following the incubation of *S*. Typhimurium with phagocytes. Indeed, we observed 3.82 folds induction of malate synthase following the incubation of *S*. Typhimurium with neutrophils. Upregulation of glyoxylate cycle genes following phagocytosis have been observed in fungal pathogens like *Paracoccidioides brasiliensis*^31^, *Penicillium marneffei*^32^, *Aspergillus fumigatus*^33^, *Cryptococcus neoformans*^34^ and *Candida albicans*^35^ as well as in several bacterial pathogens like *P. aeruginosa*^36^, *M. tuberculosis* (Munoz-Elias and McKinney 2005) and *R. equi*^30^. The microorganisms entrapped within the precarious niche of the phagolysosomes require to utilize available carbon sources for their survival under nutrient limiting and other stress conditions. Phagocytes are rich in fatty acids which might induce the activation of the enzymes of the glyoxylate cycle in the phagosome^37^. A study suggested the import of fatty acids derived from host triacylglyceraldehydes (TAGs) in *M. tuberculosis*^38^. Further, in *S*. Typhimurium, the genes for lipid metabolism and glyoxylate cycle are shown to be essential for colonization in mouse tissues like caecum, Peyer’s patches, mesenteric lymph nodes, spleen, and liver^39^. In a co-culture experiment with macrophages, the genes for lipid import, β-oxidation, and glyoxylate cycle were found to be necessary for the survival of *S*. Typhimurium^39^. Thus, an intriguing possibility of the generation of simple compounds within phagocytes may be furnished by the breakdown of fatty acids via β-oxidation, which results in acetyl-CoA, a substrate for glyoxylate cycle^10^.

In chicken, extensive bacterial multiplication takes place in the caecum^40^. After that, the bacteria reach the reticuloendothelial system^41^ and eventually to the spleen, liver, and bone marrow. Earlier, we have demonstrated that malate synthase contributes to the colonization of *S.* Typhimurium in poultry caecum^9^. Spleen and liver are the important organs involved in immune responses against *S.* Typhimurium^41^. It has been suggested that replication of *S.* Typhimurium in the SCV of a single phagocyte in either the liver or spleen is limited during acute infection^42^, however, the bacterial numbers in the liver increases by spreading from one to nearby other phagocytes, thereby forming a focus of infection^43^. Thus, although *S*. Typhimurium grows poorly in infected phagocytes, it grows in high numbers in the numerous foci of the liver^43^. These observations suggest that the metabolic substrate availability in the phagosome may evolve over the course of infection, with an increasing dependence on fatty acid and acetate utilization occurring during chronic infection. *S*. Typhimurium modulates splenic functions in ways such that the spleen serves as a safe haven for this bacterium^44,45^. Next, we assessed the contribution of malate synthase in the survival of *S.* Typhimurium in poultry spleen and liver. The dissemination of Δ*ms* strain to the spleen (*p* < 0.001) and liver (*p* < 0.01) was lower than that of the WT strain of *S.* Typhimurium (Figs. 4 and 5). The involvement of *icl* as a virulence determinant has been observed in various pathogenic organisms like *M. tuberculosis* (Munoz-Elias and McKinney 2005), *R. equi*^30^ and *P. aeruginosa*^36^. In *S.* Typhimurium, *icl* is required for chronic infection but not for acute lethal infection in mice^20^.

To survive within the host, bacterial pathogens including *S.* Typhimurium must have a series of stress management response systems. The existence of several metabolic pathways provides metabolic flexibility which helps in the utilization of various metabolites/ substrates available at a particular niche. It has been shown that the complete tricarboxylic acid (TCA) cycle operates during infection of mice with *S*. Typhimurium^46^. The glycolytic pathway is required for intracellular replication of *S.* Typhimurium in mice and macrophages and that glucose is the major sugar utilized by *S*. Typhimurium during infection of macrophages^47^. Thus, to replenish glucose and other TCA cycle intermediates during nutrient limiting and stress conditions, the glyoxylate cycle plays a pivotal role to provide the necessary energy required to replicate and survive within the host.

These observations suggest that metabolic substrate availability in the host may evolve over the course of infection, with an increasing dependence on fatty acid and acetate utilization occurring during chronic infection. Specifically, these enzymes could add potential metabolic flexibility to redox metabolism by effectively decoupling catabolic carbon flow from NADPH formation that would occur in parallel with tricarboxylic acid cycle. Therefore, in the case of *S.* Typhimurium, the central enzymes of the glyoxylate cycle might be required during different stages to accomplish its metabolic and energy requirements^48^ and aid in its survival under oxidative stress conditions.

## Experimental procedures

### Ethical statement

All animal experiments were approved by the Institutional Animal Ethics Committee (IAEC), Indian Council of Agricultural Research-Indian Veterinary Research Institute (ICAR-IVRI), Izatnagar, India with the approval file No. F.1.53/2012-13-J.D.(Res). All animal experimentations were performed in accordance with the guidelines and regulations of IAEC, ICAR-IVRI, Izatnagar, India.

### Bacterial strains and culture media

*Salmonella enterica* subspecies *enterica* serovar Typhimurium strain 5591 (*S*. Typhimurium), a poultry isolate, was procured from the repository of National *Salmonella* Centre-Veterinary, Indian Veterinary Research Institute (IVRI), Izatnagar, India. *ms* gene deletion mutant strain in *S.* Typhimurium (∆*ms* strain) was cured as described earlier^9^ and confirmed by PCR. Bacteriological media like Luria Bertani (LB) agar, LB broth, and Hektoen Enteric agar (HEA) were procured from HiMedia Laboratories Pvt. Ltd., Mumbai, India. Hank’s Balanced Salt Solution (HBSS) was prepared as per standard protocol^49^.

### In vitro growth analysis of *S. *Typhimurium and ∆*ms* strains in LB broth and M9 media

Isolated colonies of *S.* Typhimurium and ∆*ms* strains were inoculated in 10 ml of LB broth and grown at 37 °C with shaking at 180 rpm. Overnight grown cultures were diluted (@ 1:100) in fresh 50 ml of LB media and grown on a shaker incubator. Aliquots were withdrawn at every one hour of interval and the optical densities were measured at 600 nm.

The growth of *S.* Typhimurium and Δ*ms* strains were also assessed in M9 minimal media supplemented with acetate or glucose as sole carbon source. Briefly, overnight LB grown cultures were pelleted and washed with M9 minimal media. The washed pellets were then grown in M9 media supplemented with 0.4% acetate or 0.4%glucose.

### Evaluation of susceptibilities of *S. *Typhimurium and ∆*ms* strains to HOCl

Overnight grown cultures of *S.* Typhimurium and ∆*ms* strains were exposed to various concentrations of HOCl (sodium hypochlorite, NaOCl, Sigma). Following 2 h of incubation at 37 °C/ 180 rpm, the cultures were serially diluted and plated on HEA plates. Colonies were enumerated following overnight incubation of the plates.

### Evaluation of the susceptibilities of *S. *Typhimurium and ∆*ms* strains to neutrophils

Neutrophils were isolated as described elsewhere^50^ with minor modifications. The susceptibility assays were conducted according to the protocol of Okamura and Spitznagel^51^ with minor modifications. In brief, the blood was collected from the jugular vein of healthy adult goats in EDTA coated vacutainer. The neutrophils were isolated by a double density centrifugation method using Histopaque 1119/1077 (Sigma-Aldrich, USA). The cells were washed twice with phosphate-buffered saline and once with HBSS devoid of Ca^2+^/ Mg^2+^ salts (HBSS −) at 250×*g* for 10 min. The total numbers of viable cells were enumerated by Trypan blue dye exclusion method and adjusted to a concentration of 2 × 10^6^ cells/ ml using HBSS (–) media. The mid-log grown cultures of *S.* Typhimurium and ∆*ms* strains were pelleted, washed, and suspended in HBSS. The neutrophil and bacterial suspensions were mixed at a multiplicity of infection (MOI) of 1:10 (cell:bacteria). The mixture was incubated for 15 min at 37 °C and 5% CO_2_in a humidified chamber. To determine the actual MOI, the bacterial suspensions were serially diluted and plated on agar media. Following 15 min of neutrophil- bacterial incubation, the suspensions were centrifuged at 13,000 rpm for 3 min. The supernatant was discarded and the pellet was lysed by 0.1% Triton X-100 for 5 min. Lysates were serially diluted and plated on HEA plates. Colonies were enumerated following overnight incubation of the plates.

### Expression analysis of *ms* in *S.* Typhimurium

The expression analysis of the *ms*gene was carried out by qRT-PCR. Neutrophils were infected with *S.* Typhimurium for 15 min as described in the above section. RNA from the harvested pellet was isolated by Trizol reagent. RNA isolated from LB broth grown cultures was served as a control. qRT- PCR was performed according to the protocol as described in Maxima H Minus First Strand cDNA Synthesis Kit (Thermo Scientific). In brief, for a 20 μl reaction, 500 ng of RNA was mixed with 1 μl of random primer (100 pmol), 1 μl of dNTP mix (10 mM), 4 μl of 5× RT buffer, and 1 μl of Maxima H Minus enzyme mix. cDNA was synthesized by incubation of the above mix at 25 °C for 10 min followed by 50 °C for 15 min and termination at 85 °C for 5 min. The expression levels of *ms* gene in each sample were assessed and normalized using DNA gyrase B (*gyrB*) as an internal control^52^. For a 20 μl of qRT-PCR reaction, 0.5 μM of each primer, 2 μl of cDNA, and 10 μl of SYBR Green (Thermo Scientific) were used. The relative fold change in gene expression was determined using the 2^−ΔΔCT^ method^53^. The initial denaturation was carried out at 95 °C for 5 min, followed by 40 cycles consisting of denaturation at 95 °C for 10 s, annealing at 62 °C for 30 s, and data acquisition at 74 °C for 30 s. The amplified products were analyzed on 1.5% agarose gel for assessment of nonspecific amplification (if any) and primer dimers.

### Assessment of the contribution of malate synthase in the colonization of *S.* Typhimurium in poultry spleen and liver

All experiments were carried out according to the guidelines of the Institute’s Animal Ethics Committee, ICAR- IVRI, Izatnagar, India and in accordance with the ARRIVE guidelines. The bacterial burdens in the liver and spleen were assessed as described earlier^54^. In brief, one-day-old chicks were procured from ICAR-Central Avian Research Institute (CARI), Izatnagar, India, and provided with ad libitum feed and water. The birds were screened for the presence of *Salmonella* spp. The *Salmonella* free birds were divided into two groups and orally infected with *S.* Typhimurium or Δ*ms* strain. Following 7, 14, and 21 days post-infection, 5 birds were sacrificed from each group.100 mg of the liver and whole spleen were homogenized in 1 ml of PBS. 100 µl of homogenized samples were plated on HEA plates. The plates were incubated overnight at 37 °C.

### Oxyblot analysis

Oxidation status of the malate synthase was assessed by OxyBlot™ Protein Oxidation Detection Kit (EMD Millipore). Malate synthase was purified and incubated with 100 mM H_2_O_2_ as described earlier^9^. Excess H_2_O_2_ was removed by dialysis. Varying concentrations (2, 1, 0.5 μg) of the H_2_O_2_ exposed malate synthase samples were denatured with 6% SDS (final) and derivatized with 2, 4-dinitrophenyhydrazine (DNPH). The derivatized samples were then resolved on 10% SDS gel and electroblotted onto the nitrocellulose membrane. Following blocking the blot was incubated in anti-DNPH antibodies and developed as mentioned in a protocol described elsewhere^55^.

### Western blotting analysis

*S*. Typhimurium was cultured in M9 minimal media supplemented either with glucose or acetate. The late stationary phase grown cultures were pelleted and washed with ice cold PBS. The pellets were then lysed by BugBuster Master Mix (EMD Millipore Corp, USA) and unbroken cells were removed by centrifugation. Total proteins in the clarified supernatants were estimated by the Pierce BCA Protein Assay Kit (Thermo Scientific, USA). Fifteen micrograms of cell free lysates were resolved on SDS-gel and transferred to PVDF membranes. Following blocking, the membranes were incubated with anti-MS hyper immune sera (1: 50,000 dilutions in PBS-Tween 20). Following washing, the membrane was washed in anti-rabbit IgG conjugated with alkaline phosphatase (Sigma, at a dilution of 1: 15,000 in PBS-Tween 20) and developed by using NBT and BCIP as substrate^9^.

### Statistical analysis

The graphical representation of the data was done by using Microsoft Excel software and the analysis of results was done by one way analysis of variance (ANOVA).

## Supplementary Information

Supplementary Figures.

## Data Availability

All data generated or analysed during this study are included in this published article [and its supplementary information files].

## References

[1] Gal-Mor O, Boyle EC, Grassl GA (2014). Same species, different diseases: How and why typhoidal and non-typhoidal *Salmonella enterica* serovars differ. Front. Microbiol..

[2] Reddy EA, Shaw AV, Crump JA (2011). Community-acquired bloodstream infections in Africa: A systematic review and meta-analysis. Lancet Infect. Dis..

[3] Galanis E, Wong DMLF, Patrick ME, Binsztein N, Cieslik A, Chalermchaikit T, Aidara-Kane A, Ellis A, Angulo FJ, Wegener HC (2006). Web-based surveillance and global *Salmonella* distribution, 2000–2002. Emerg. Infect. Dis..

[4] Fàbrega A, Vila J (2013). *Salmonella enterica* serovar Typhimurium skills to succeed in the host: Virulence and regulation. Clin. Microbiol. Rev..

[5] Jones BBD, Ghori N, Falkow S (1994). *Salmonella* Typhimurium initiates murine infection by penetrating and destroying the specialized epithelial M Cells of the peyer ’ s patches. J. Exp. Med..

[6] Portillo FG (2001). *Salmonella* intracellular proliferation : where, when and how ?. Microbes Infect..

[7] Abrahams GL, Hensel M (2006). Manipulating cellular transport and immune responses : Dynamic interactions between intracellular *Salmonella enterica* and its host cells. Cell. Microbiol..

[8] Aussel R, Zhao W, Hébrard M, Guilhon AA, Viala JP, Henri S, Chasson L, Gorvel JP, Barras F, Méresse S (2011). Salmonella detoxifying enzymes are sufficient to cope with the host oxidative burst. Mol. Microbiol..

[9] Sarkhel R, Rajan P, Gupta AK, Kumawat M, Agarwal P, Shome A, Puii L, Mahawar M (1861). (2017) Methionine sulfoxide reductase A of *Salmonella* Typhimurium interacts with several proteins and abets in its colonization in the chicken. BBA-Gen. Subj..

[10] Lorenz MC, Fink GR (2002). Life and death in a macrophage: Role of the glyoxylate cycle in virulence. Eukaryot. cell..

[11] Kwon YM, Ricke SC (1998). Survival of a *Salmonella* Typhimurium poultry isolate in the presence of propionic acid under aerobic and anaerobic conditions. Anaerobe.

[12] Kornberg HL (1966). The role and control of the glyoxylate cycle in *Escherichia coli*. The Biochem. J..

[13] Remmerie A, Scott CL (2018). Macrophages and lipid metabolism. Cell. Immunol..

[14] Kornberg HL, Krebs HA (1957). Nat. Publ. Group.

[15] Campbell JJR, Smith RA, Eagles BA (1953). A deviation from the conventional tricarboxylic acid cycle in *Pseudomonas Aeruginosa*. BBA.

[16] Ajl SJ (1956). Conversion of acetate and glyoxylate to malate. J. Am. Chem. Soc..

[17] Dunn MF, Ramírez-Trujillo JA, Hernández-Lucas I (2009). Major roles of isocitrate lyase and malate synthase in bacterial and fungal pathogenesis. Microbiology.

[18] Rui B, Shen T, Zhou H, Liu J, Chen J, Pan X, Liu H, Wu J, Zheng H, Shi Y (2010). A systematic investigation of *Escherichia coli* central carbon metabolism in response to superoxide stress. BMC Syst. Biol..

[19] Singh KS, Sharma R, Keshari D, Singh N, Singh SK (2017). Down-regulation of malate synthase in *Mycobacterium tuberculosis* H37Ra leads to reduced stress tolerance, persistence and survival in macrophages. Tuberculosis.

[20] Fang FC, Libby SJ, Castor ME, Fung AM (2005). Isocitrate lyase (AceA) is required for *Salmonella* persistence but not for acute lethal infection in mice. Infect. Immun..

[21] Dalle-Donne I, Rossi R, Giustarini D, Milzani A, Colombo R (2003). Protein carbonyl groups as biomarkers of oxidative stress. Clin. Chim. Acta..

[22] Wilson RB, Maloy SR (1987). Isolation and characterization of Salmonella typhimurium glyoxylate shunt mutants. J. Bacteriol. Res..

[23] Ahn S, Jung J, Jang IA, Madsen EL, Park W (2016). Role of glyoxylate shunt in oxidative stress response. J. Biol. Chem..

[24] Nandakumar M, Nathan C, Rhee KY (2014). Isocitrate lyase mediates broad antibiotic tolerance in *Mycobacterium tuberculosis*. Nat. Commun..

[25] Jung J, Park W (2013). Comparative genomic and transcriptomic analyses reveal habitat differentiation and different transcriptional responses during pectin metabolism in *Alishewanella*species. Appl. Environ. Microbiol..

[26] Jung J, Noh J, Park W (2011). Physiological and metabolic responses for hexadecane degradation in *Acinetobacter oleivorans*DR1. J. Microbiol..

[27] Fridovich I (1983). Superoxide radical:an endogenous toxicant. Annu. Rev. Pharmacol. Toxicol..

[28] Brumaghim JL, Li Y, Henle E, Linn S (2003). Effects of hydrogen peroxide upon nicotinamide nucleotide metabolism in *Escherichia coli*: Changes in enzyme levels and nicotinamide nucleotide pools and studies of the oxidation of NAD(P)H by Fe(III). J. Biol. Chem..

[29] Ha S, Shin B, Park W (2018). Lack of glyoxylate shunt dysregulates iron homeostasis in *Pseudomonas aeruginosa*. Microbiology (UK).

[30] Wall DM, Duffy PS, DuPont C, Prescott JF, Meijer WG (2005). Isocitrate lyase activity is required for virulence of the intracellular pathogen *Rhodococcusequi*. Infect. Immun..

[31] Derengowski LS, Tavares AH, Silva S, Procópio LS, Felipe MSS, Silva-Pereira I (2008). Upregulation of glyoxylate cycle genes upon *Paracoccidioides brasiliensis* internalization by murine macrophages and in vitro nutritional stress condition. Med. Mycol..

[32] Thirach S, Cooper CR, Vanittanakom N (2008). Molecular analysis of the *Penicillium marneffei* glyceraldehyde-3-phosphate dehydrogenase-encoding gene (*gpdA*) and differential expression of *gpdA*and the isocitrate lyase-encoding gene (*acuD*) upon internalization by murine macrophages. J. Med. Microbiol..

[33] Ebel F, Schwienbacher M, Beyer J, Heesemann J, Brakhage AA, Brock M (2006). Analysis of the regulation, expression, and localisation of the isocitrate lyase from *Aspergillus fumigatus*, a potential target for antifungal drug development. Fungal Genet. Biol..

[34] Fan W, Kraus PR, Boily MJ, Heitman J (2005). *Cryptococcus neoformans* gene expression during murine macrophage infection. Eukaryot. Cell..

[35] Lorenz MC, Fink GR (2001). The glyoxylate cycle is required for fungal virulence. Nature.

[36] Lindsey TL, Hagins JM, Sokol PA, Silo-Suh LA (2008). Virulence determinants from a cystic fibrosis isolate of *Pseudomonas aeruginosa* include isocitrate lyase. Microbiology.

[37] Calder PC (2008). The relationship between the fatty acid composition of immune cells and their function. Prostag. Leukotr. Ess..

[38] Daniel J, Maamar H, Deb C, Sirakova TD, Kolattukudy PE (2011). *Mycobacterium tuberculosis* uses host triacylglycerol to accumulate lipid droplets and acquires a dormancy-like phenotype in lipid-loaded macrophages. PlosPathog..

[39] Reens AL, Nagy TA, Detweiler CS (2020). *Salmonella enterica* requires lipid metabolism genes to replicate in proinflammatory macrophages and mice. Infect. Immun..

[40] Barrow PA, Simpson JM, Lovell MA (1988). Intestinal colonisation in the chicken by food-poisoning *Salmonella* serotypes; microbial characteristics associated with faecal excretion. Avian Pathol..

[41] Coburn B, Grassl GA, Finlay BB (2007). *Salmonella*, the host and disease: A brief review. Immunol. Cell Biol..

[42] Salcedo SP, Noursadeghi M, Cohen J, Holden DW (2001). Intracellular replication of *Salmonella* Typhimurium strains in specific subsets of splenic macrophages *in vivo*. Cell. Microbiol..

[43] Sheppard M, Webb C, Heath F, Mallows V, Emilianus R, Maskell D, Mastroeni P (2003). Dynamics of bacterial growth and distribution within the liver during *Salmonella* infection. Cell. Microbiol..

[44] Rosche KL, Aljasham AT, Kipfer JN, Piatkowski BT, Konjufca V (2015). Infection with *Salmonellaenterica* Serovar Typhimurium leads to increased proportions of F4/80+ red pulp macrophages and decreased proportions of B and T lymphocytes in the spleen. PLoS ONE.

[45] Dunlap NE, Benjamin WH, McCall RD, Tilden AB, Briles DE (2006). A ‘safe-site’ for Salmonella typhimurium is within splenic cells during the early phase of infection in mice. Microb. Pathog..

[46] Yimga MT, Leatham MP, Allen JH, Laux DC, Conway T, Cohen PS (2006). Role of gluconeogenesis and the tricarboxylic acid cycle in the virulence of *Salmonella enterica* serovar Typhimurium in BALB/c mice. Infect. Immun..

[47] Bowden SD, Rowley G, Hinton JCD, Thompson A (2009). Glucose and glycolysis are required for the successful infection of macrophages and mice by *Salmonella enterica* serovar Typhimurium. Infect. Immun..

[48] Diacovich L, Lorenzi L, Tomassetti M, Meresse S, Gramajo H (2017). The infectious intracellular lifestyle of *Salmonella enterica* relies on the adaptation to nutritional conditions within the Salmonella-containing vacuole. Virulence..

[49] Hanks JH, Wallace RE (1949). Relation of oxygen and temperature in the preservation of tissues by refrigeration. Proc. Soc. Exp. Biol. Med..

[50] Oh H, Siano B, Diamond S (2008). Neutrophil isolation protocol. J. Vis. Exp..

[51] Okamura N, Spitznagel JK (1982). Outer membrane mutants of *Salmonella* Typhimurium LT2 have lipopolysaccharide-dependent resistance to the bactericidal activity of anaerobic human neutrophils. Infect. Immun..

[52] Lamichhane-Khadka R, Frye JG, Porwollik S, McClelland M, Maier RJ (2011). Hydrogen-stimulated carbon acquisition and conservation in *Salmonella enterica* serovar typhimurium. J. Bacteriol..

[53] Pfaffl MW (2001). A new mathematical model for relative quantification in real-time RT-PCR. Nucleic Acids Res..

[54] Pesingi PK, Kumawat M, Behera P, Dixit SK, Agarwal RK, Goswami TK, Mahawar M (2017). Protein-L-isoaspartyl methyltransferase (PIMT) is required for survival of *Salmonella* Typhimurium at 42°C and contributes to the virulence in poultry. Front. Microbiol..

[55] Apoorva S, Behera P, Sajjanar B, Mahawar M (2020). Identification of oxidant susceptible proteins in *Salmonella* Typhimurium. Mol. Biol. Rep..

[56] Chung T, Klumpp DJ, LaPorte DC (1988). Glyoxylatebypass operon of *Escherichia coli*: Cloning and determination of the functional map. J. Bacteriol..

[57] Cozzone AJ (1998). Regulation of acetate metabolism by protein phosphorylation in enteric bacteria. Annu. Rev. Microbiol..

[58] Krebs HA (1937). The intermediate metabolism of carbohydrates. Lancet.

[59] Muñoz-Elías EJ, McKinney JD (2005). *Mycobacterium tuberculosis* isocitrate lyases 1 and 2 are jointly required for in vivo growth and virulence. Nat. Med..

[60] Zheng J, Yates SP, Jia Z (2012). Structural and mechanistic insights into the bifunctional enzyme isocitrate dehydrogenase kinase/phosphatase AceK. Philos. Trans. R. Soc. B. Biol. Sci..

