# Supplementary material for: Malate synthase contributes to the survival of Salmonella Typhimurium against nutrient and oxidative stress conditions
Source: Sci Rep. 2022 Sep 25;12:15979. doi: 10.1038/s41598-022-20245-0 (PMC9510125; doi:10.1038/s41598-022-20245-0)
Supplement: Supplementary file 1 — Supplementary Figures. [file 41598_2022_20245_MOESM1_ESM.pptx]

## Slide 1
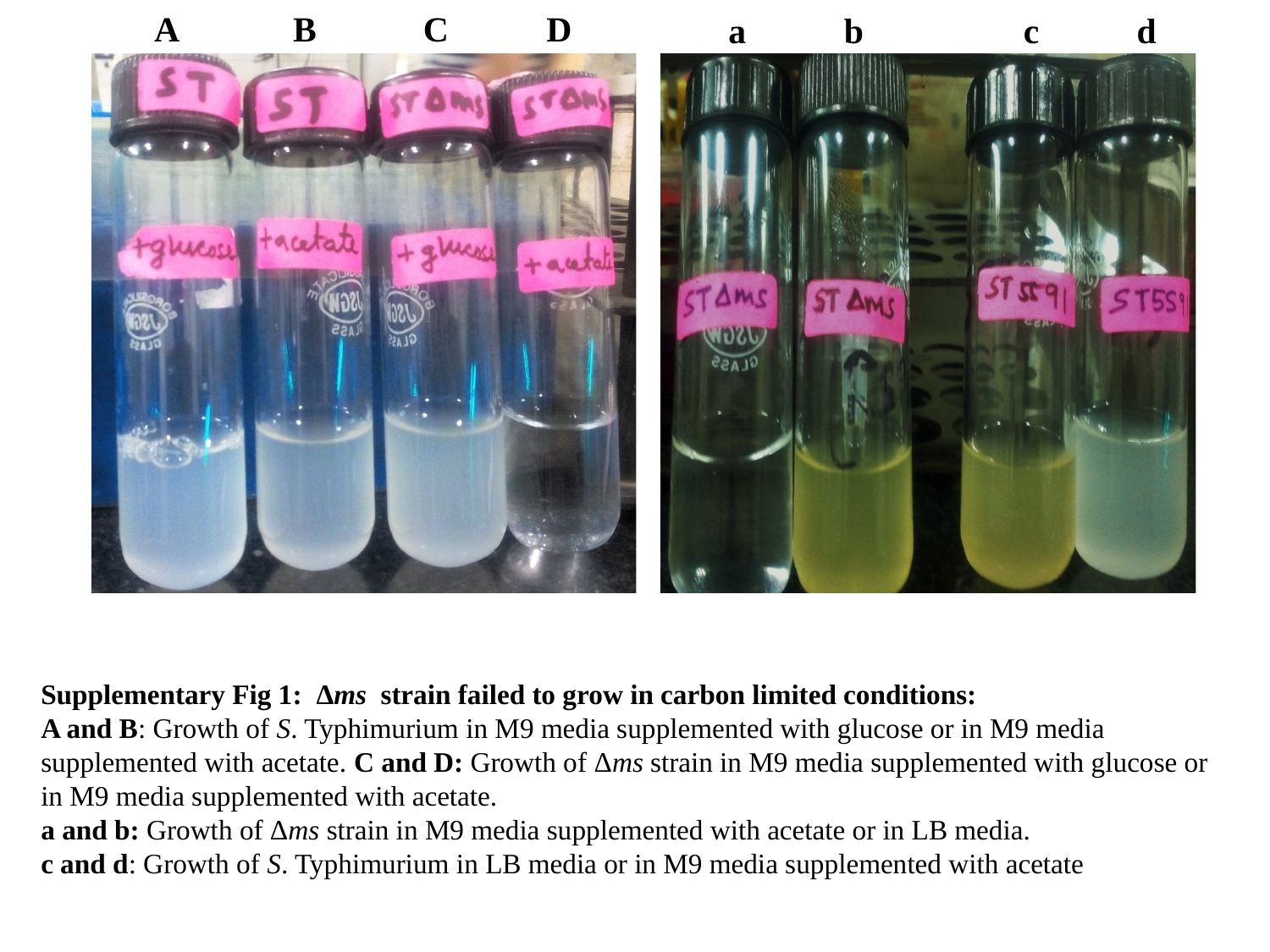

A B C D
 a b c d
# Supplementary Fig 1: Δms strain failed to grow in carbon limited conditions: A and B: Growth of S. Typhimurium in M9 media supplemented with glucose or in M9 media supplemented with acetate. C and D: Growth of Δms strain in M9 media supplemented with glucose or in M9 media supplemented with acetate. a and b: Growth of Δms strain in M9 media supplemented with acetate or in LB media. c and d: Growth of S. Typhimurium in LB media or in M9 media supplemented with acetate

## Slide 2
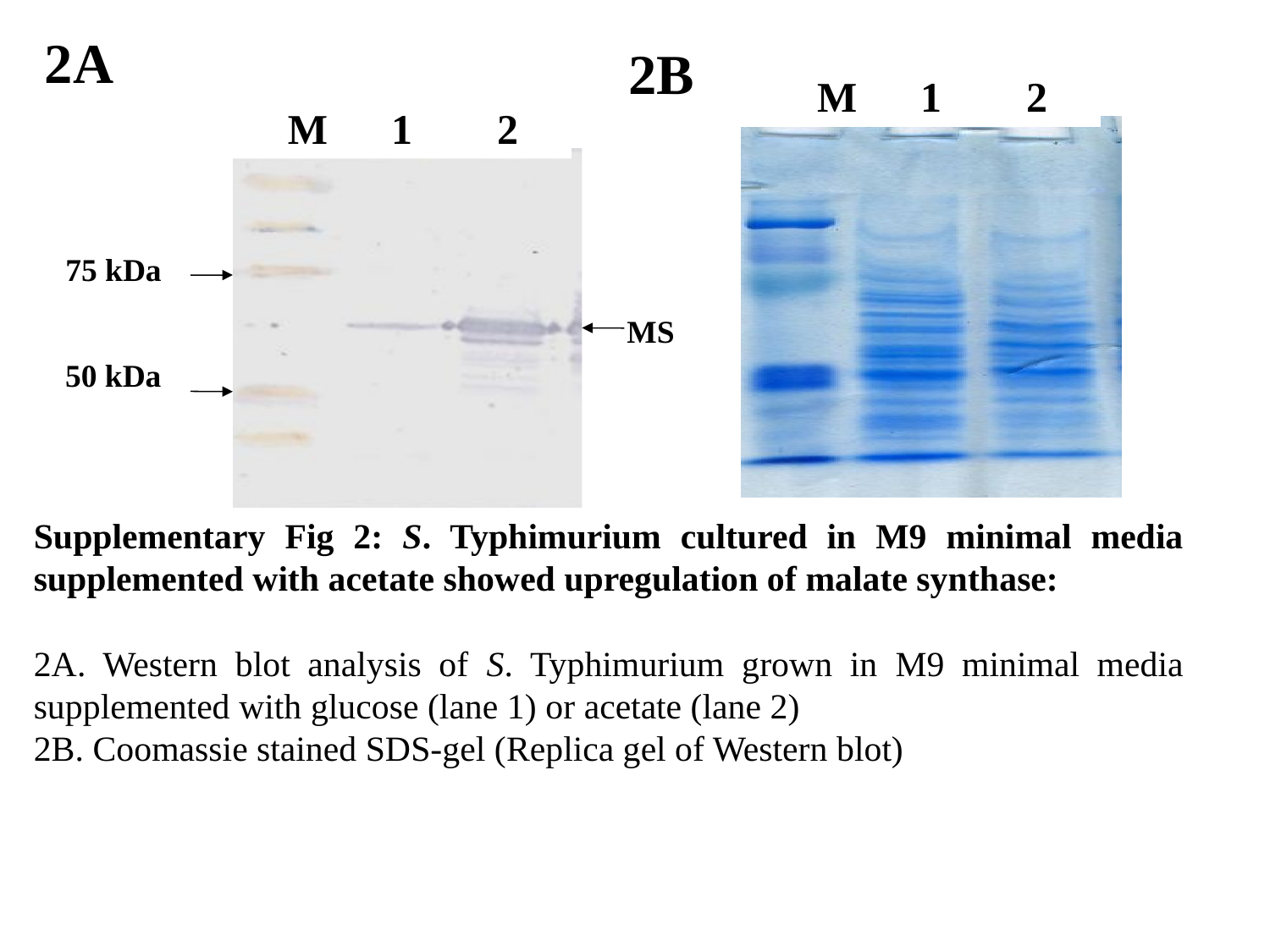

2A
2B
 M 1 2
 M 1 2
75 kDa
MS
50 kDa
Supplementary Fig 2: S. Typhimurium cultured in M9 minimal media supplemented with acetate showed upregulation of malate synthase:
2A. Western blot analysis of S. Typhimurium grown in M9 minimal media supplemented with glucose (lane 1) or acetate (lane 2)
2B. Coomassie stained SDS-gel (Replica gel of Western blot)

## Slide 3
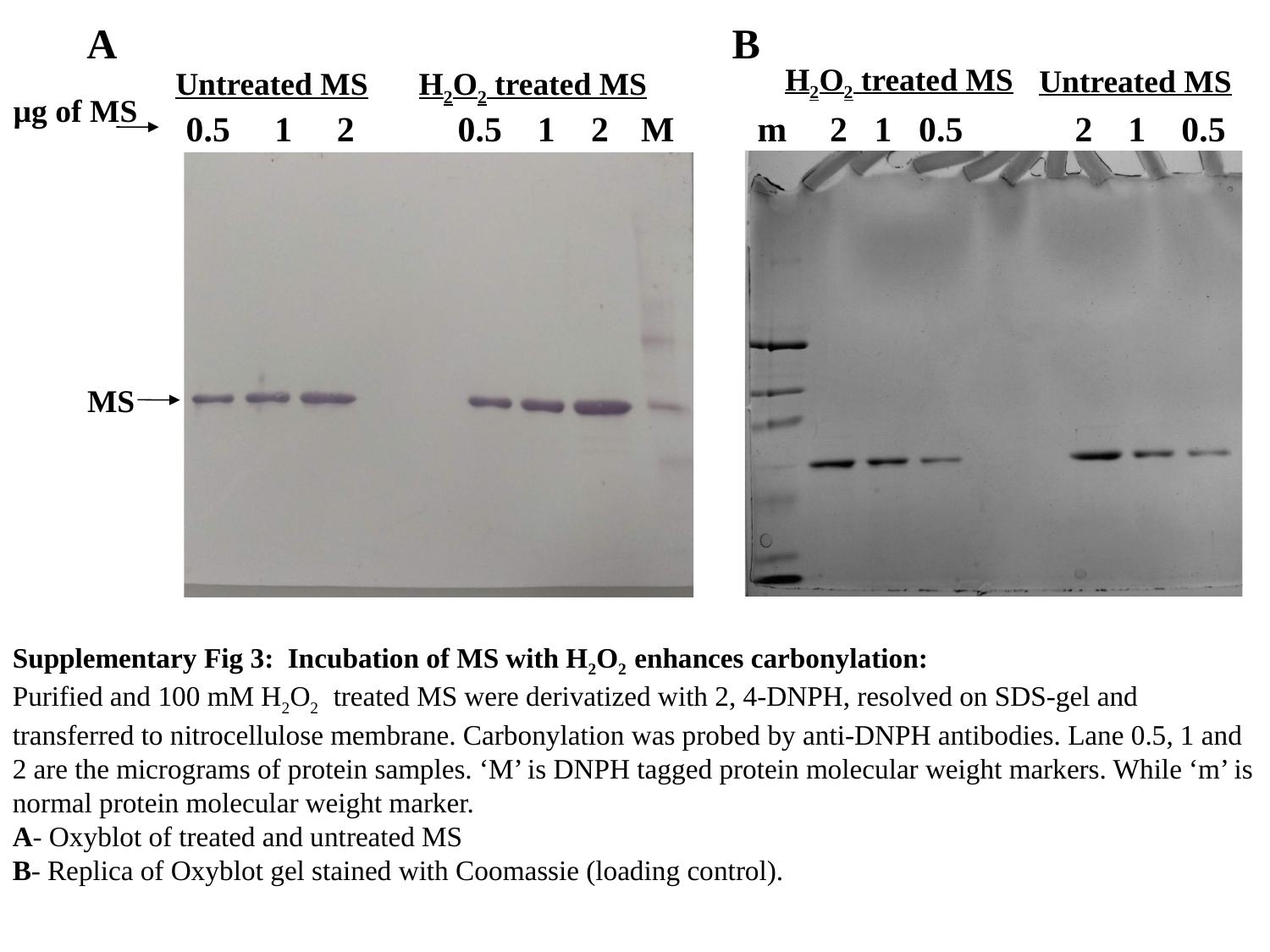

A
B
H2O2 treated MS
Untreated MS
Untreated MS
H2O2 treated MS
µg of MS
0.5 1 2
 0.5 1 2
M
m
 2 1 0.5
2 1 0.5
MS
Supplementary Fig 3: Incubation of MS with H2O2 enhances carbonylation:
Purified and 100 mM H2O2 treated MS were derivatized with 2, 4-DNPH, resolved on SDS-gel and transferred to nitrocellulose membrane. Carbonylation was probed by anti-DNPH antibodies. Lane 0.5, 1 and 2 are the micrograms of protein samples. ‘M’ is DNPH tagged protein molecular weight markers. While ‘m’ is normal protein molecular weight marker.
A- Oxyblot of treated and untreated MS
B- Replica of Oxyblot gel stained with Coomassie (loading control).

## Slide 4
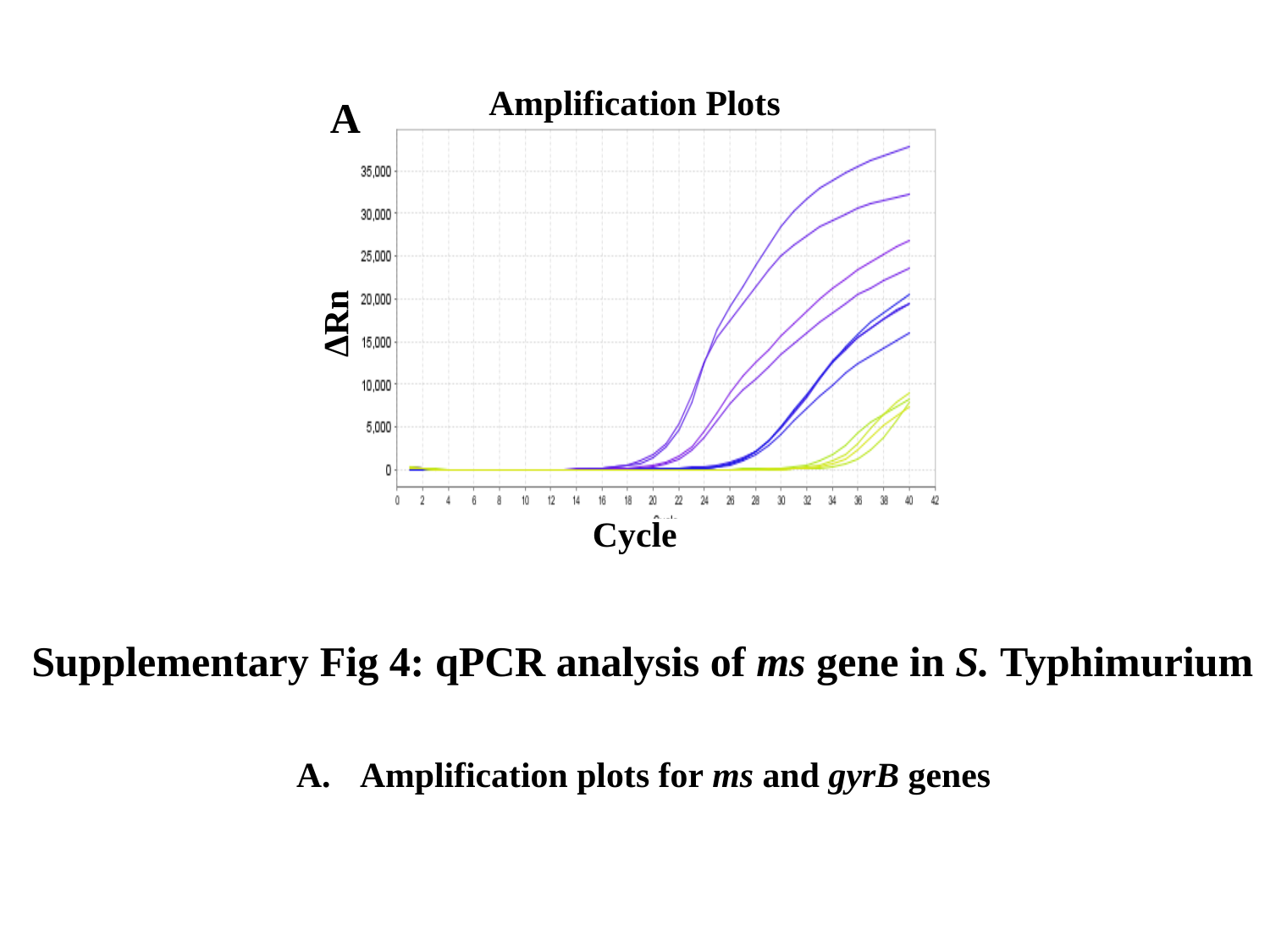

Amplification Plots
A
ΔRn
Cycle
Supplementary Fig 4: qPCR analysis of ms gene in S. Typhimurium
Amplification plots for ms and gyrB genes

## Slide 5
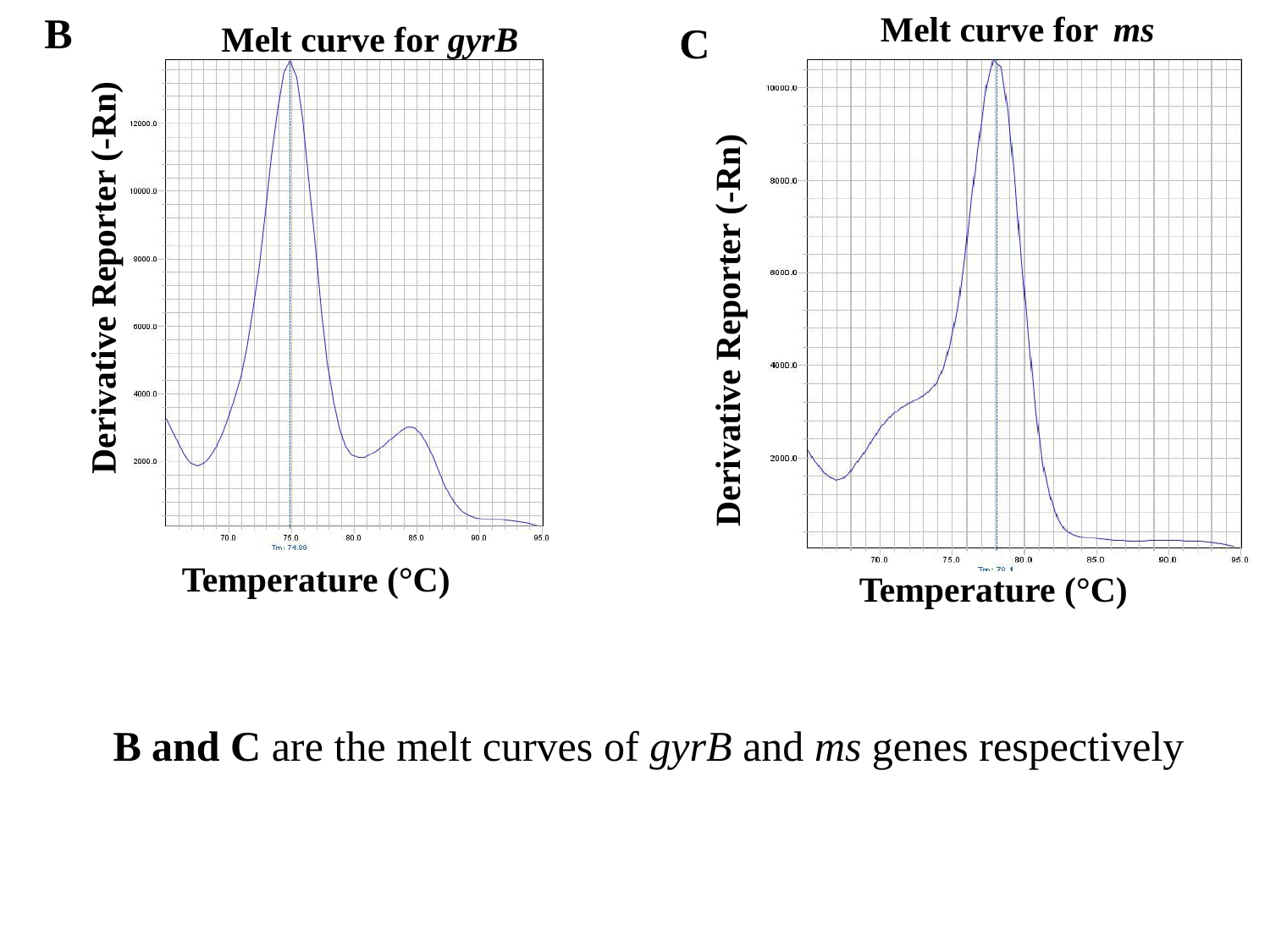

B
Melt curve for
ms
Melt curve for gyrB
C
Derivative Reporter (-Rn)
Derivative Reporter (-Rn)
Temperature (°C)
Temperature (°C)
B and C are the melt curves of gyrB and ms genes respectively

## Slide 6
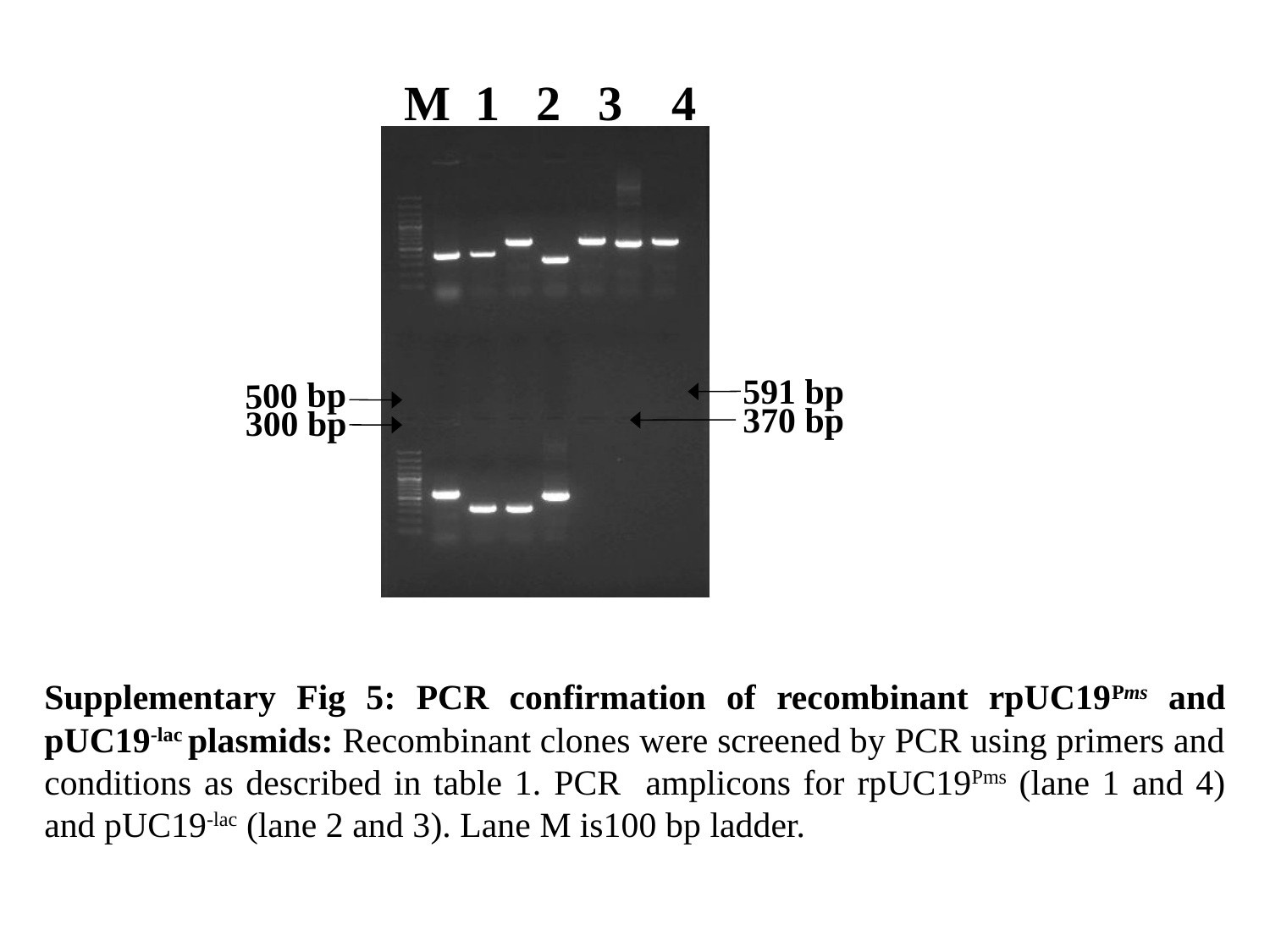

M 1 2 3 4
591 bp
500 bp
370 bp
300 bp
Supplementary Fig 5: PCR confirmation of recombinant rpUC19Pms and pUC19-lac plasmids: Recombinant clones were screened by PCR using primers and conditions as described in table 1. PCR amplicons for rpUC19Pms (lane 1 and 4) and pUC19-lac (lane 2 and 3). Lane M is100 bp ladder.

## Slide 7
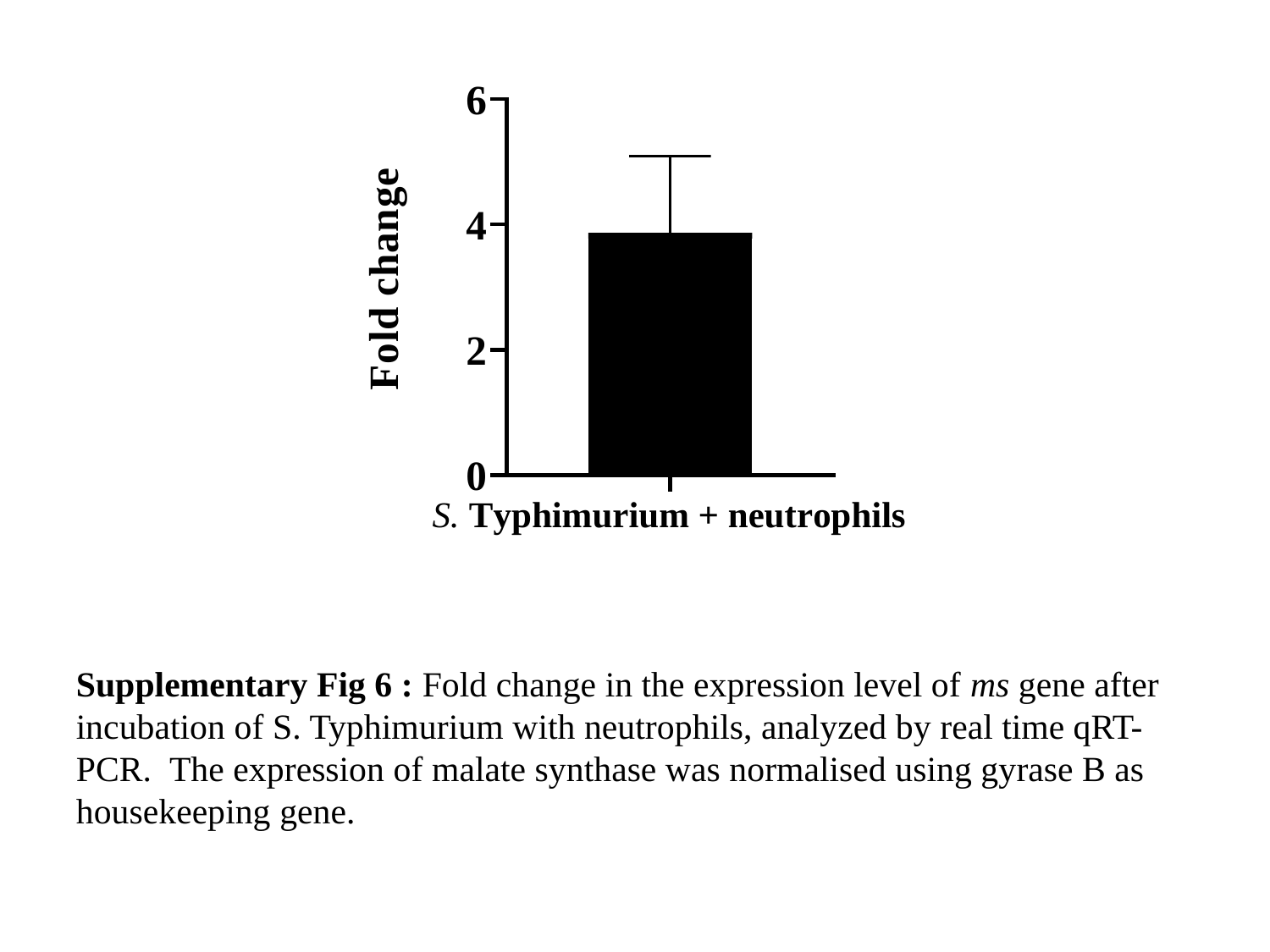

Supplementary Fig 6 : Fold change in the expression level of ms gene after incubation of S. Typhimurium with neutrophils, analyzed by real time qRT-PCR. The expression of malate synthase was normalised using gyrase B as housekeeping gene.
